# CXXC5 Attenuates Pulmonary Fibrosis in a Bleomycin-Induced Mouse Model and MLFs by Suppression of the CD40/CD40L Pathway

**DOI:** 10.1155/2020/7840652

**Published:** 2020-04-04

**Authors:** Wei Cheng, Fangfang Wang, Airan Feng, Xiaodan Li, Wencheng Yu

**Affiliations:** ^1^Department of Respiratory and Critical Care Medicine, The Affiliated Hospital of Qingdao University, No. 16 Jiangsu Road, Qingdao, 266000 Shandong, China; ^2^Department of Respiratory Medicine, The Fifth Hospital of Xiamen City, No. 101 Min'an Road, Xiamen, 361101 Fujian, China; ^3^Department of Postgraduate, Qingdao University, No. 308 Ningxia Road, Qingdao, 266071 Shandong, China

## Abstract

**Objective:**

To investigate the role of CXXC5 and the CD40/CD40L pathway in lung fibrosis.

**Methods:**

(1) We constructed mouse models of bleomycin-induced pulmonary fibrosis and transfected them with a CXXC5 overexpression vector to evaluate the severity of pulmonary fibrosis. (2) Mouse lung fibroblast (MLF) models stably overexpressed or knockout of CXXC5 vector were constructed. After transforming growth factor-*β*1 (TGF-*β*1) stimulation, we examined the proliferation and apoptosis of the MLF model and evaluated the expression of mesenchymal markers and the CXXC5/CD40/CD40L pathway.

**Results:**

(1) Compared with other groups, the overexpressed CXXC5 group had less alveolar structure destruction, thinner alveolar septum, and lower Ashcroft score. (2) In bleomycin-induced mice, the expression of CD40 and CD40L increased at both transcriptional and protein levels, and the same changes were observed in *α*-smooth muscle actin (*α*-SMA) and collagen type I (Colla I). After upregulation of CXXC5, the increase in CD40, CD40L, *α*-SMA, and Colla I was attenuated. (3) Stimulated with TGF-*β*1, MLF proliferation was activated, apoptosis was suppressed, and the expression of CD40, CD40L, *α*-SMA, and Colla I was increased at both transcriptional and protein levels. After upregulation of CXXC5, these changes were attenuated.

**Conclusion:**

CXXC5 inhibits pulmonary fibrosis and transformation to myofibroblasts by negative feedback regulation of the CD40/CD40L pathway.

## 1. Introduction

Idiopathic pulmonary fibrosis (IPF) is a chronic progressive fibrosis disease, which is the most common idiopathic interstitial pneumonia (IIP). Repeated microinjury and abnormal repair of alveolar epithelial cells and activation of fibroblasts are the key factors to the pathogenesis of IPF [1]. The prognosis of IPF is poor, and no effective treatment was found currently, of which the median survival after diagnosis is only 2-3 years. In 2014, the US Food and Drug Administration- (FDA-) approved nintedanib [2] and pirfenidone [3] may slow down the decline in lung function in patients with IPF but cannot change the IPF survival rate. Lung transplantation is the only treatment for patients with end-stage pulmonary fibrosis. However, the lung source is limited, and the immune rejection after transplantation is difficult to treat.

CXXC5 is a retinoid-responsive gene that is located in the 5q31.3 chromosomal region and encodes a retinoid-inducible nuclear factor (RINF) [4], which is widely expressed in fibroblasts, bone cells, nerve cells, cardiomyocytes, stem cells, and hepatocytes [5–8]. CXXC5 was found to be downregulated in various fibrotic diseases such as scar formation and scleroderma [9]. CD40 is an integrated membrane protein of 34-39 kDa type II, which was initially found in B lymphocytes and also expressed in fibroblasts, antigen-presenting cells (APCs), epithelial cells, and vascular endothelial cells. The expression of CD40 is low in normal physiological conditions, and it can be significantly upregulated under pathological conditions [10]. Activation of CD40 and CD40L in pulmonary fibrosis causes fibroblast aggregation, proliferation, and expression of IL-6, IL-8, ICAM-1, and VCAM-1, causing collagen deposition and pulmonary interstitial changes leading to fibrosis finally. Therefore, we hypothesize that CXXC5 may participate in the development of pulmonary interstitial fibrosis through the CD40/CD40L pathway and explore the role of the CXXC5/CD40/CD40L pathway in an in vitro and in vivo fibrosis model.

## 2. Materials and Methods

### 2.1. Overexpression and Interference with the Construction, Identification, and Amplification of Adenoviral Vectors

Tianjin Saier Biological Company was commissioned to construct and verify the adenoviral vectors of Ad-CXXC5, Ad-shR-CXXC5, and Ad-GFP with a green fluorescent protein tracer gene.

### 2.2. Construction of Bleomycin-Induced Fibrosis Mice and Collection of Lung Tissue Samples

Animal studies were conducted following the guidelines of the Experimental Animal Ethics Committee of the Affiliated Hospital of Qingdao University. All experimental procedures were approved by the Experimental Animal Ethics Committee of the Affiliated Hospital of Qingdao University. Male C57BL/6 mice weighing 18-22 g were provided by the Institute of Experimental Animals of Chinese Academy of Medical Sciences (Beijing, China) which were divided into 6 groups, 15 in each group. We designed the following six groups in the experiment: (1) control group (MOCK group), (2) model group (BLM group), (3) saline+Ad-GFP group (MOCK+Ad-GFP group), (4) saline+AdCXXC5 group (MOCK+Ad-CXXC5 group), (5) bleomycin+Ad-GFP group (BLM+Ad- GFP group), and (6) bleomycin+Ad-CXXC5 group (BLM+Ad-CXXC5 group). Bleomycin (Sigma, USA) was dissolved in sterile saline at a concentration of 1 mg/mL. In BLM, BLM+Ad-CXXC5, and BLM+Ad-GFP groups, bleomycin solution (2 mg/kg) was injected into the trachea in the inspiratory phase and the same volume of aseptic saline was injected into the MOCK group, MOCK+Ad-GFP group, and MOCK+Ad-CXXC5 group in the same way. On the second day of modeling, the mice in each group were injected with the corresponding adenoviruses (5 × 10^9^ PFU/mouse) into the tail vein. The mice were closely observed and were euthanized at 7 days, 14 days, and 28 days after modeling of 5 mice separately. The fresh right lung tissue specimens were immediately placed in 4% paraformaldehyde for hematoxylin and eosin staining (HE staining kit C0105, Biyuntian Company, China) and Masson staining (kit C0105, Biyuntian Company, China). The left lung tissue was divided into two parts and stored in a refrigerator at -80°C immediately. Hydroxyproline was tested according to the instructions of the hydroxyproline test kit (A0303, Nanjing Jiancheng Company, China), and another copy was extracted with RNA and cryopreserved according to the protocol of the RNA extraction kit (1406120, Ambion, USA).

### 2.3. Culture and Identification of Mouse Lung Fibroblast Cells (MLFs)

Eight-week-old C57BL/6 mice were selected and sacrificed by cervical dislocation, and 1 × 1 × 1 mm lung tissue was obtained and cultured in Dulbecco's Modified Eagle's Medium (DMEM) containing 10% calf serum (Sigma, USA) in a 5% CO_2_, fully humidified 37°C incubator. The solution was changed every 3 to 4 days. When the cells reached 80% fusion, they were digested with 0.25% trypsin and sorted by a differential attachment method. MLFs in a logarithmic growth phase were inoculated into a 24-well plate with slides and cultured for 18-24 hours. After adding cells with 4% paraformaldehyde for 30 min, Triton X-100 was permeabilized for 5 min, vimentin antibody primary antibody binding solution diluted with 1% donkey serum (Sigma, USA) was added, and the second antibody solution (FITC-labeled donkey anti-rabbit IgG secondary antibody) and DAPI cell staining solution were added the next day; then, the slides in the well plate were taken out and buckled on a slide containing a fluorescent protective agent and observed under a fluorescence microscope.

### 2.4. Determination of Optimal Infection Efficiency of Ad-CXXC5, Ad-GFP, and Ad-ShRNA-CXXC5 Adenoviruses

The optimal TGF-*β*1 concentration for stimulating cells was 10 ng/mL. The lung fibroblasts in the logarithmic growth phase were selected and inoculated into a 48-well plate, and the cell density was about 60-70% the next day. The lung fibroblasts in the logarithmic growth phase were infected with different multiplicity of infection of viruses (3 gradient multiplicity of infection (MOI) values per virus: 50/100/200) in complete cell culture medium. In view of that, the following experiments were all detected 48 hours after infection; therefore, the expression of green fluorescent protein was observed under a fluorescence microscope and photographed after 48 hours of infection.

### 2.5. Detection of Cell Proliferation and Apoptosis

#### 2.5.1. Cell Proliferation Assay

The Cell Counting Kit-8 (CCK-8) (Ameresco, USA) was used to measure cell proliferation according to the instructions, and each set of experiments was repeated 3 times. Annexin-V-PE/flow cytometry with an apoptosis detection kit (Shanghai Biyuntian Company, China) was used to measure the apoptotic rate: taking 50,000-100,000 cell suspension, centrifuged at 1000g for 5 min and added with 200 *μ*L V-FITC binding solution. Then, the cells were resuspended, added with 10 *μ*L of propidium iodide (PI) staining solution, incubated for 10-20 minutes in the dark, and tested on a flow cytometer.

### 2.6. Detection of mRNAs of CXXC5, CD40, CD40L, *α*-SMA, and Colla I in Animals and Cells by Real-Time Quantitative Polymerase Chain Reaction (RT-qPCR)

(1) Total RNA was extracted, and purity and concentration were determined in cells and animal lung tissues in each group; (2) cDNA was obtained by reverse transcription; (3) fluorescence quantitative PCR amplification of the target gene was performed, and *β*-actin (internal reference) was used as a control; and (4) data processing and analysis were performed, and a column chart was made.  Table 1 shows the primer design and synthesis.

### 2.7. Detection of the Expression of CXXC5, CD40, CD40L, *α*-SMA, and Colla I in Cells and Lung Tissues of Mice by Western Blot

Total protein of MLFs and lung tissues were collected by using RIPA lysate buffer, and the protein concentration of each sample was determined by the BCA method. Then, SDS-PAGE was performed and the proteins were transferred from the gel onto PVDF membranes. Following blocking with 2% BSA, the membranes were incubated with primary and secondary antibodies. The LabWorks™ gel imaging and analysis system (UVP, USA) was used for photography. The band brightness of the samples to be tested was compared with the relative *β*-actin (internal reference) luminance value, and the ratio obtained was plotted after standardization. Anti-CXXC5, anti-*α*-SMA, anti-Colla I, anti-CD40L (PE), anti-CD40 (FITC) antibodies, etc. were all purchased from Abcam Company of the United States.

### 2.8. Statistical Analysis

SPSS 22.0 statistical software (IBM Corp., Armonk, NY, USA) was used for analysis. Experimental data between two groups were compared with the *t*-test, the data of each group was expressed as mean ± standard deviation, and the difference was statistically significant at *P* < 0.05.

## 3. Results

### 3.1. Overexpression of CXXC5 Attenuated Pulmonary Fibrosis in Mice

**Figure 1:**
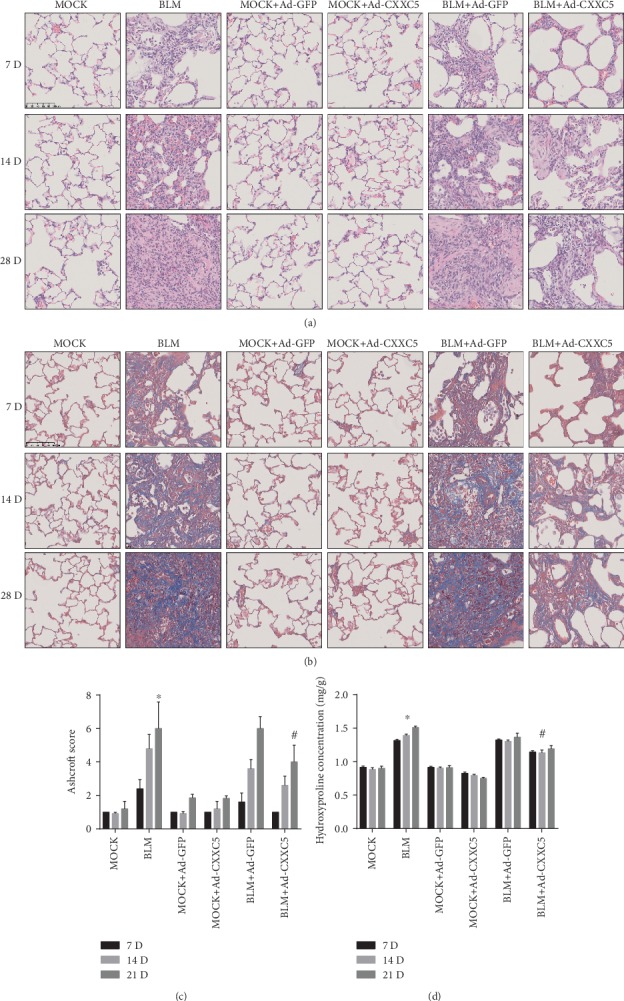
(a) HE staining of mice in each group at three time points (200x). (b) Masson staining at three time points in each group (200x). (c) Pulmonary fibrosis score at three time points in each group. (d) Hydroxyproline content at three time points in each group of lung tissues (^∗^*P* < 0.05 compared with MOCK group; ^#^*P* < 0.05 compared with BLM group or BLM+Ad-GFP group).

Compared with the MOCK group, the alveolar structure of the BLM group was severely damaged and the alveolar space was widened (Figure 1(a)). The blue-stained collagen fibers were significantly increased (Figure 1(b)), and the pulmonary fibrosis score (Figure 1(c)) and hydroxyproline (Figure 1(d)) content were increased. And these indicators gradually increased over time, reaching the highest level at day 28. There was no difference in the pulmonary fibrosis score and hydroxyproline content between the BLM+Ad-GFP group and BLM group as well as between the MOCK+Ad-GFP group and MOCK group. The BLM+Ad-GFP group suffered from more severe alveolar structural damage (Figure 1(a)) and had more collagen fibers (Figure 1(b)) and higher pulmonary fibrosis score (Figure 1(c)) and hydroxyproline (Figure 1(d)) content than the BLM+Ad-CXXC5 group, and the difference was statistically significant (*P* < 0.05). It was confirmed that overexpression of CXXC5 inhibited BLM-induced pulmonary fibrosis.

### 3.2. CXXC5 Overexpression Inhibited CD40/CD40L Pathway Activation

**Figure 2:**
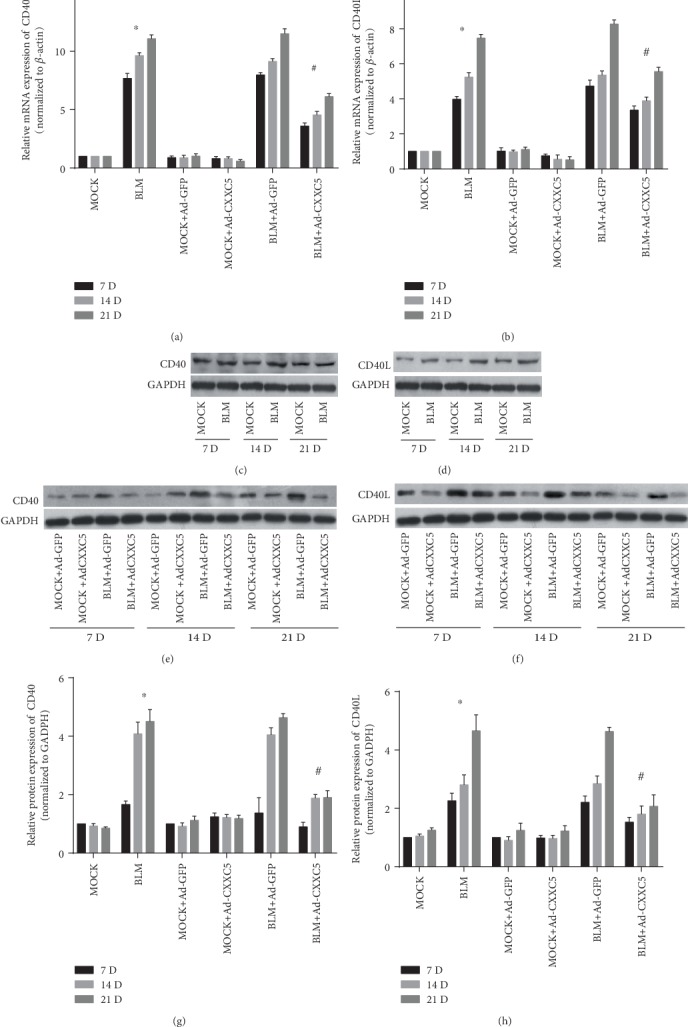
(a, b) The mRNA expression of CD40 and CD40L in the lung tissues of each group at 7, 14, and 28 days. (c–f) The protein expression of CD40 and CD40L in lung tissues of each group measured by Western blot at 7, 14, and 28 days. (g, h) The relative protein expression of CD40 and CD40L in lung tissues of each group at 7, 14, and 28 days by Western blot (^∗^*P* < 0.05 compared with MOCK group; ^#^*P* < 0.05 compared with BLM group or BLM+Ad-GFP group).

Compared with the MOCK group, the BLM group had higher CD40 and CD40L mRNA (Figures 2(a) and 2(b)) and protein levels (Figures 2(c)–2(d), 2(g), and 2(h)) at the same time points on days 7, 14, and 28. The highest level was found at day 28 after model establishment, and the difference was statistically significant (*P* < 0.05). There was no difference in mRNA and protein levels of CD40 and CD40L in the BLM+Ad-GFP group and BLM group as well as the MOCK+Ad-GFP group and MOCK group. Compared with the BLM+Ad-GFP group, the CD40 and CD40L mRNA (Figures 2(a) and 2(b)) and protein (Figures 2(e)–2(h)) in the BLM+Ad-CXXC5 group were significantly decreased and the difference was statistically significant (*P* < 0.05). Thus, CXXC5 overexpression inhibited the activation of the CD40/CD40L signaling pathway in a mouse model of pulmonary fibrosis.

### 3.3. Effects of CXXC5 on Mesenchymal Markers and the Extracellular Matrix in Mice with Pulmonary Fibrosis

**Figure 3:**
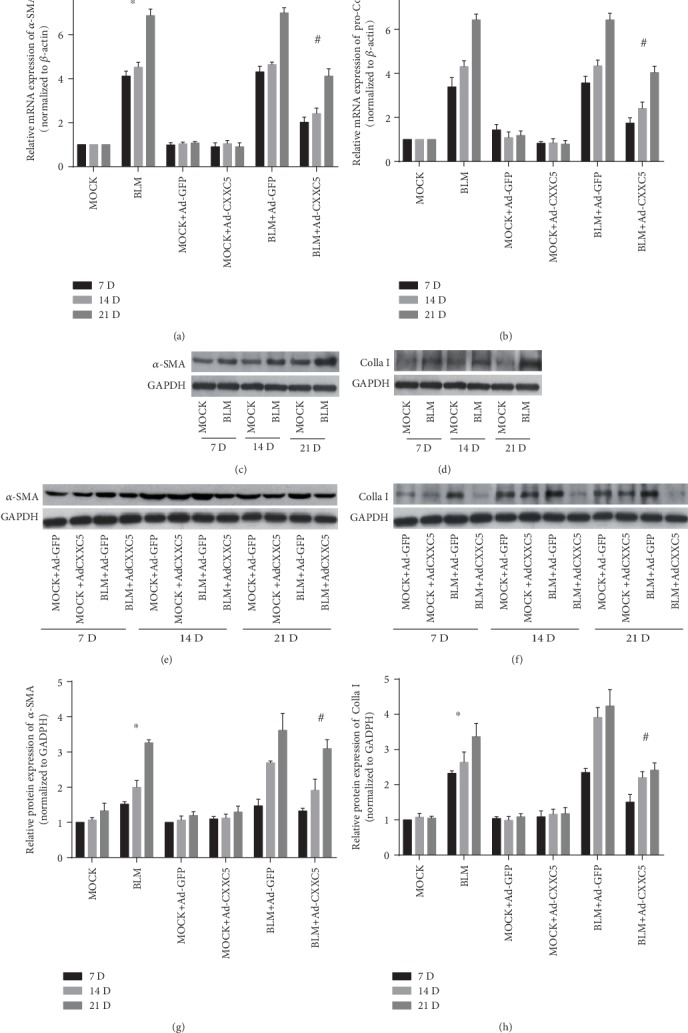
(a, b) The mRNA expression of *α*-SMA and pro-Colla I in the lung tissue of each group at 7, 14, and 28 days. (c–f) Western blot was used to measure the protein expression of *α*-SMA and Colla I in the lung tissues of each group at 7, 14, and 28 days. (g, h) The relative protein expression of *α*-SMA and Colla I in the lung tissues of each group was measured by Western blot (^∗^*P* < 0.05 compared with MOCK group; ^#^*P* < 0.05 compared with BLM group or BLM+Ad-GFP group (*P* < 0.05)).

On days 7, 14, and 28, the mRNAs of *α*-SMA and pro-Colla I (Figures 3(a) and 3(b)) in the BLM group were significantly higher than those in the MOCK group (*P* < 0.05), and the same changes were observed at the protein level (Figures 3(c), 3(d), 3(g), and 3(h)). There was no significant difference in mRNA and protein levels of *α*-SMA and Colla I between the BLM+Ad-GFP group and BLM group as well as between the MOCK+Ad-GFP group and MOCK group. Compared with the BLM+Ad-CXXC5 group, the mRNAs of *α*-SMA and pro-Colla I (Figures 3(a) and 3(b)) in the BLM+Ad-GFP group were significantly increased and so did the protein level (Figures 3(e)–3(h)). The difference was significant (*P* < 0.05). Thus, CXXC5 overexpression can inhibit the expression of *α*-SMA and Colla I in pulmonary fibrosis.

### 3.4. Isolation and Culture of MLFs and Construction of Ad-CXXC5, Ad-GFP, and Ad-shRNA-CXXC5 Stable Strains

**Figure 4:**
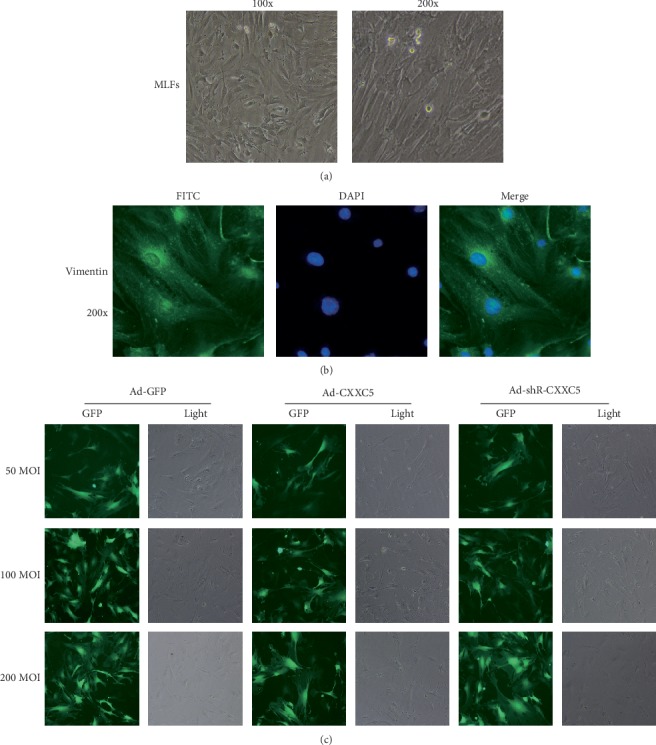
(a) The morphology of MLFs under an inverted microscope (100x, 200x). (b) Identification of isolated and cultured MLFs by immunofluorescence, and vimentin was strongly positive (200x). (c) Screening of the best MOI for MLFs (100 MOI was the best).

Mouse lung fibroblasts (MLFs) were isolated by a differential attachment method and identified in two ways. One is morphological observation, and MLFs were spindle-shaped and strip-like under an inverted microscope (Figure 4(a)). On the other hand, we tested vimentin immunofluorescence in MLFs because it was strongly expressed in the cytoplasm of MLFs (Figure 4(b)). The above results all confirmed that the cultured cells in our experiment were MLFs.

The optimal infection efficiency (100 MOI) of Ad-CXXC5, Ad-GFP, and Ad-shRNA-CXXC5 adenoviruses was screened, and the stable strain of MLFs was constructed (Figure 4(c)). After successful construction, the cell experiments were divided into 8 groups: (1) Cell group, (2) Ad-GFP group, (3) Ad-CXXC5 group, and (4) Ad-shR-CXXC5 group and after TGF-*β*1 stimulation (5) TGF-*β*1 group, (6) TGF-*β*1+Ad-GFP group, (7) TGF-*β*1+Ad-CXXC5 group, and (8) TGF-*β*1+Ad-shR-CXXC5 group.

### 3.5. CXXC5 Inhibited the Activation of MLFs into Myofibroblasts

**Figure 5:**
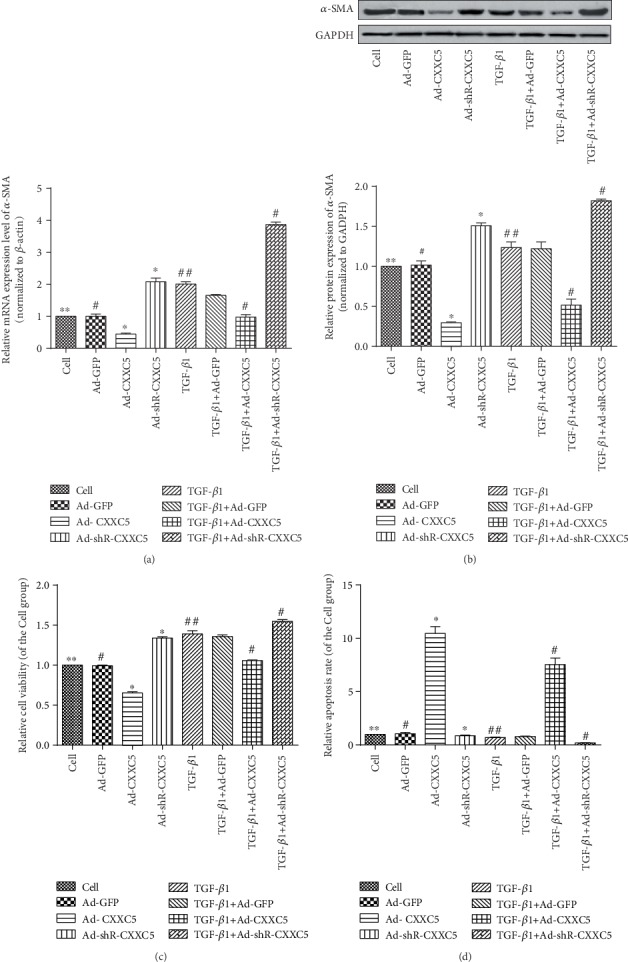
(a) *α*-SMA mRNA in each group of cells. (b) *α*-SMA protein in each group of cells. (c) Proliferation of each group of cells. (d) Apoptosis of each group of cells (^∗^*P* < 0.05 compared with Ad-GFP group; ^∗∗^*P* > 0.05 compared with Ad-GFP group; ^#^*P* < 0.05 compared with TGF-*β*1+Ad-GFP group; ^##^*P* > 0.05 compared with TGF-*β*1+Ad-GFP group).

When CXXC5 was overexpressed, the mRNA and protein of *α*-SMA were decreased in fibroblasts; when CXXC5 was underexpressed, the mRNA and protein of *α*-SMA in fibroblasts increased (Figures 5(a) and 5(b)) (*P* < 0.05). Compared with the Ad-GFP group, the proliferation of Ad-CXXC5 cells was decreased (*P* < 0.05), but apoptosis was significantly increased (*P* < 0.05), suggesting that CXXC5 inhibited the proliferation of MLFs and promoted apoptosis. The proliferation of the Ad-shR-CXXC5 group was significantly higher than that of the Ad-GFP group (*P* < 0.05), and the apoptosis was significantly decreased (*P* < 0.05), suggesting that downregulating CXXC5 promoted cell proliferation (Figure 5(c)) and inhibited apoptosis (Figure 5(d)).

Then, we stimulated MLFs with TGF-*β*1. There was no significant difference in the proliferation and apoptosis rate between the TGF-*β*1 group and TGF-*β*1+Ad-GFP group. Compared with the TGF-*β*1+Ad-GFP, the proliferation of the TGF-*β*1+Ad-CXXC5 group was significantly lower and the apoptosis rate was significantly higher (*P* < 0.05), while the proliferation activity of TGF-*β*1+Ad-shR-CXXC5 was significantly increased and the apoptosis rate was significantly decreased (*P* < 0.05) (Figures 5(c) and 5(d)). Therefore, CXXC5 can inhibit the proliferation of MLFs induced by TGF-*β*1 and promote apoptosis.

### 3.6. CXXC5 Overexpression Inhibited TGF-*β*1-Induced Activation of the CD40/CD40L Signaling Pathway

**Figure 6:**
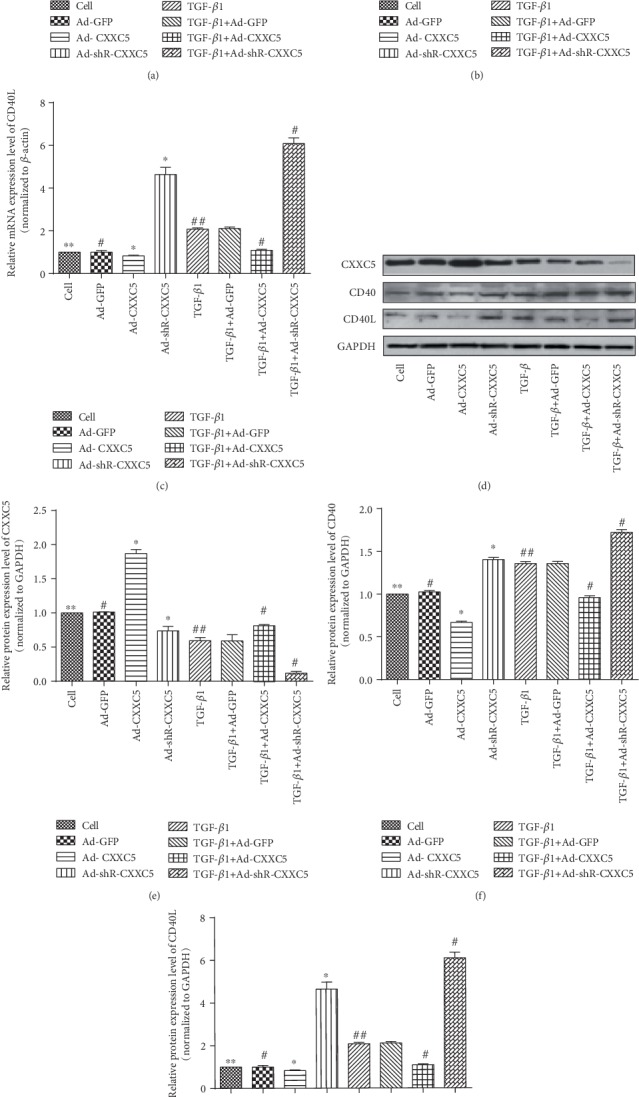
(a–c) The mRNA expression of CXXC5, CD40, and CD40L in each group. (d) Western blot measured the protein expression of CXXC5, CD40, and CD40L in each group. (e–g) The relative protein expression of CXXC5, CD40, and CD40L in each group (^∗^*P* < 0.05 compared with Ad-GFP group; ^∗∗^*P* > 0.05 compared with Ad-GFP group; ^#^*P* < 0.05 compared with TGF-*β*1+Ad-GFP group; ^##^*P* > 0.05 compared with TGF-*β*1+Ad-GFP group).

We cultured MLFs in vitro and performed cell grouping and treatment as above, and RT-qPCR and Western blot were used to detect CXXC5. We found that the mRNA and protein expression of CXXC5 in the TGF-*β*1 group was significantly lower than that in the Cell group (*P* < 0.05) (Figures 6(a), 6(d), and 6(e)). Therefore, we considered that TGF-*β*1 inhibited the expression of CXXC5 in mouse lung fibroblasts.

CD40 and CD40L mRNA (Figures 6(b) and 6(c)) and protein (Figures 6(d) and 6(f)–6(g)) in the TGF-*β*1 group were significantly increased than those in the Cell group. They were decreased in fibroblasts when CXXC5 was overexpressed. When CXXC5 was knocked out, CD40 and CD40L mRNA and protein levels were increased. Therefore, CXXC5 can inhibit the activation of the CD40/CD40L signaling pathway induced by TGF-*β*1.

## 4. Discussion

Idiopathic pulmonary fibrosis (IPF) is a chronic progressive lung disease that is the most common and the worst prognostic type of interstitial lung disease (ILD). The alveolar epithelium is damaged in IPF, which also secretes fiber growth factors such as TGF-*β*1, osteopontin, periostin, connective tissue growth factor (CTGF), and fibronectin [11, 12]. Fibroblast proliferation and activation for myofibroblasts cause excessive deposition of extracellular matrix (ECM) [13, 14] structural destruction and respiratory failure. The prognosis of IPF is poor, and there is currently no effective treatment. Lung transplantation is the only treatment for end-stage patients.

CXXC5 is a gene located in the region of the 5q31.3 chromosome, also known as RINF, which encodes a vitamin C-inducible nuclear protein containing the CXXC zinc finger group [15]. There are few studies on pulmonary fibrosis in CXXC5 locally and abroad, and the role and mechanism of CXXC5 in pulmonary fibrosis are unclear. CXXC5 was first found in myeloid leukemia [16, 17], and later, CXXC5 overexpression was found in breast cancer, prostate cancer [18, 19], and other malignant diseases. Besides, CXXC5 overexpression was associated with the poor prognosis of malignant tumors. Therefore, CXXC5 is considered to be a tumor inhibitor, involved in apoptosis, but the specific mechanism is unclear [20]. In the study of skin fibrosis in mice, it was found that CXXC5 played an important role, and CXXC5 protein decreased in the acute trauma of keratinocytes and fibroblasts. Overexpression of CXXC5 inhibited the transformation of mouse skin fibroblasts into myofibroblasts and the synthesis of keratin 14 and collagen, which slowed down the healing of the mouse skin [9]. By RT-PCR and Western blot, we found that CXXC5 expression was low in the bleomycin-induced pulmonary fibrosis model and in vitro pulmonary fibrosis model, showing that CXXC5 was involved in pulmonary fibrosis.

We successfully constructed the CXXC5 overexpression group through an adenoviral vector and further studied the role of CXXC5 in the progress of pulmonary fibrosis. By comparing HE staining, Masson staining, hydroxyproline content, and pulmonary fibrosis score in lung tissue, we identified that the BLM+Ad-CXXC5 group had significantly reduced alveolar damage collagen deposition compared with the BLM group. At the same time, overexpression of CXXC5 reduced the content of hydroxyproline in lung tissue and decreased the score of pulmonary fibrosis, indicating that CXXC5 can inhibit the progression of pulmonary fibrosis and has an antifibrotic effect.

In the formation of pulmonary fibrosis, damaged type II alveolar epithelial cells cannot be effectively repaired and induce local fibroblasts to transform into myofibroblasts [21]. Myofibroblasts can upregulate the expression of extracellular matrix proteins such as Colla I and *α*-SMA which are specific markers of myofibroblasts. Myofibroblasts aggregate into fiber foci and continuously produce ECM. A large number of ECM deposits lead to the loss of alveolar structure and function, resulting in the formation of fibrosis [22]. We compared the expressions of *α*-SMA and Colla I in lung tissue of each group at transcriptional and protein levels. The expression of *α*-SMA and Colla I was downregulated in the BLM+Ad-CXXC5 group compared with the BLM group. In order to verify that CXXC5 is involved in the transformation of fibroblasts into myofibroblasts, we designed to upregulate and downregulate CXXC5 and detected the expression of Colla I and *α*-SMA by RT-PCR and Western blot. We found that *α*-SMA and Colla I decreased after CXXC5 upregulation with or without TGF-*β*1 stimulation. After downregulation of CXXC5, *α*-SMA and Colla I were all increased. Therefore, we consider that CXXC5 can inhibit the transformation of fibroblasts into myofibroblasts.

We used the CCK-8 method to measure the proliferative activity of cells and measured the apoptosis rate of cells by flow cytometry. The effect of CXXC5 on MLFs and the effect of CXXC5 and TGF-*β*1 on the activity of MLFs were determined. In the absence of TGF-*β*1 stimulation, CXXC5 inhibited the proliferation activity of MLFs and promoted apoptosis. After silencing the expression of CXXC5, the proliferation activity of cells increased and apoptosis was inhibited. When MLFs were stimulated with TGF-*β*1, CXXC5 inhibited the proliferation activity of MLFs and promoted apoptosis, while after silencing CXXC5, the proliferation activity was increased and apoptosis was inhibited. Therefore, CXXC5 can inhibit the proliferation and apoptosis of MLFs and can also inhibit the proliferation and apoptosis of fibroblasts induced by TGF-*β*1.

CD40 and CD40L belong to the cytokines and receptors in the tumor necrosis factor (TNF) family, respectively. CD40/CD40L was initially considered to be an immunoregulatory factor involved in the activation of immune cells, but later, CD40 was found to be expressed in other nonhematopoietic cells, including lung fibroblasts [18, 19], indicating that CD40/CD40L may regulate the function of fibroblasts. CD40 on the surface of fibroblasts can bind to CD40L, promote the proliferation of fibroblasts and possibly the production of IL-6 and IL-8 by activating the NF-*κ*B pathway, and express ICAM and VCAM-1. IL-4 has a synergistic effect with the ligands of CD40 which can jointly promote the proliferation of fibroblasts [22–24]. Studies have shown that blocking the CD40/CD40L pathway attenuated radiation-induced oxidative stress and immune-induced lung damage and pulmonary fibrosis. We conducted further studies to explore whether CXXC5 exerted antifibrotic effects through the negative regulation of CD40/CD40L and found that CD40 and CD40L were upregulated in the pulmonary fibrosis model both in vitro and in vivo, but CD40 and CD40L were downregulated after CXXC5 overexpression. Based on the results of cell and animal experiments, we found a significant increase in CD40 and CD40L in the model of in vitro and in vivo pulmonary fibrosis and a significant decrease in CD40 and CD40L when CXXC5 was overexpressed. Therefore, we conclude that overexpression of CXXC5 inhibits the development of pulmonary fibrosis by downregulating the CD40/CD40L pathway.

Our experiment demonstrated that CXXC5 inhibited MLF proliferation and transformation into myofibroblasts via the CD40/CD4L pathway and promoted apoptosis of MLFs. Overexpression of CXXC5 inhibited the progression of bleomycin-induced pulmonary fibrosis in mice by inhibiting the CD40/CD40L signaling pathway, which is possibly a new target for the treatment of pulmonary fibrosis.

## Figures and Tables

**Table 1:** Design and synthesis of primers for target genes.

Gene	Sequence
Colla I a1 forward primer	5′GAGACAGGCGAACAAGGTGA3′
Colla I a1 reverse primer	5′CTCAAGGTCACGGTCACGAA3′
*α*-SMA forward primer	5′GTACCCAGGCATTGCTGACA3′
*α*-SMA reverse primer	5′GAGGCGCTGATCCACAAAAC3′
CD40L forward primer	5′TTGTTGACAGCGGTCCATCT3′
CD40L reverse primer	5′TGAATGGGCGTTGACTCGAA3′
CD40 forward primer	5′TTGTTGACAGCGGTCCATCT3′
CD40 reverse primer	5′TCTCAAGAGCTGTGCAGTGG3′
CXXC5 forward primer	5′GTCCGAGCAGAGCCAGAAG3′
CXXC5 reverse primer	5′CGGCTGCCCACAATAGAGAT3′
*β*-Actin forward primer	5′CCACCATGTACCCAGGCATT3′
*β*-Actin reverse primer	5′CGGACTCATCGTACTCCTGC3′

Abbreviations: Colla I a1: collagen type I alpha 1; *α*-SMA: *α*-smooth muscle actin.

## Data Availability

All data included in this study are available upon request by contact with the corresponding author.
